# Risk factors for nonvisualization of the sentinel lymph node on lymphoscintigraphy in breast cancer patients

**DOI:** 10.1186/s13550-021-00793-8

**Published:** 2021-06-09

**Authors:** Youssef Chahid, Xinbo Qiu, Ewoudt M. W. van de Garde, Hein J. Verberne, Jan Booij

**Affiliations:** 1 Department of Radiology and Nuclear Medicine, Amsterdam University Medical Centers, University of Amsterdam, Amsterdam, the Netherlands; 2 Department of Clinical Pharmacy, Amsterdam University Medical Centers, University of Amsterdam, Amsterdam, the Netherlands; 3 Department of Clinical Pharmacy, St. Antonius Hospital, Utrecht/Nieuwegein, the Netherlands; 4 Division of Pharmacoepidemiology and Clinical Pharmacology, Department of Pharmaceutical Sciences, Utrecht University, Utrecht, the Netherlands

**Keywords:** Lymphoscintigraphy, Nonvisualization, Sentinel lymph node, Breast cancer

## Abstract

**Background:**

Accurate sentinel lymph node (SLN) staging is essential for both prognosis and treatment in patients with breast cancer. However, the preoperative lymphoscintigraphy may fail to visualize the SLN in some patients. The purpose of this retrospective study was to identify risk factors associated with SLN nonvisualization on lymphoscintigraphy. For this single-center retrospective study, all data of lymphoscintigraphy of SLN procedures from March 2011 to April 2021 were collected and reviewed from the Amsterdam UMC database.

**Results:**

A total of 1886 SLN procedures were included in this study. The SLN nonvisualization rate was 25.1% on lymphoscintigraphy at 4 h post-injection. The SLN nonvisualization rate decreased to 9.4% after reinjection. Multivariable analysis showed that age ≥ 70 years (*P* < 0.001; OR: 2.27; 95% CI: 1.46–3.53), BMI ≥ 30 kg/m^2^ (*P* = 0.031; OR: 1.48; 95% CI: 1.04–2.12) and nonpalpable tumors (*P* = 0.004; OR: 1.54; 95% CI: 1.15–2.07) were independent predictors of SLN nonvisualization. Tumor location, brand of radiopharmaceutical, injected dose and volume, experience of preparer and administrator were not associated with SLN nonvisualization. None of the patient, tumor or tracer characteristics were associated with SLN nonvisualization after radiotracer reinjection.

**Conclusions:**

This study shows that risk factors for SLN nonvisualization in breast cancer patients during preoperative lymphoscintigraphy are age ≥ 70 years, BMI ≥ 30 kg/m^2^ and nonpalpable tumors. Our results support the notion that SLN lymphoscintigraphy is a very robust technique that does not depend on the experience of the preparer or administrator of the radiotracer.

**Supplementary Information:**

The online version contains supplementary material available at 10.1186/s13550-021-00793-8.

## Background

It is well known that accurate sentinel lymph node (SLN) staging is essential for both prognosis and treatment in patients with breast cancer selected to undergo the SLN procedure [1]. However, the preoperative lymphoscintigraphy may fail to visualize the SLN in some patients. In the literature, reported rates of SLN nonvisualization vary between 2 and 28% [2–7]. Different patient characteristics (body mass index (BMI), age) and tumor characteristics (size, location, palpability) have been found to be associated with SLN nonvisualization [2–7].

Our nuclear medicine physicians claimed that a change of radiotracer had led to an increased SLN nonvisualization rate. However, information of radiotracer characteristics on SLN nonvisualization is limited. Although the injected dose of the radiotracers and injection technique seem to be correlated with SLN non-visualization [2], information on the potential impact of the level of experience in, for example, radiotracer preparation or the level of experience of the administrator is absent.

Therefore, the purpose of this retrospective study was to validate the claim of the our nuclear medicine physicians that a change of radiotracer had led to an increased SLN nonvisualization rate. In doing so, we enlarged the scope of the of the study by also trying to identify potential unknown independent factors associated with SLN nonvisualization on lymphoscintigraphy.

## Methods

### Patient population and data extraction

This single-center retrospective study was approved by the local Medical Ethics Review Committee of the Academic Medical Center (AMC), Amsterdam, the Netherlands. Lymphoscintigraphy data of SLN procedures from March 2011 to April 2021 were collected and reviewed from the Amsterdam UMC, location AMC, database. We considered reinjections and bilateral procedures as separate SLN procedures. No patient was excluded.

The following data were collected from the database: age (divided into three categories: < 50 years, 50–70 years, and ≥ 70 years) [6], BMI (divided into three categories: < 30 kg/m^2^, 25–30 kg/m^2^, and ≥ 30 kg/m^2^) [7], tumor palpability (divided into two categories: palpable and nonpalpable) [6], tumor location (divided into two categories: lateral and medial/central) [6], brand of radiopharmaceutical (divided into two categories: Nanocoll, and Nanoscan). The following characteristics were divided based on the distribution of our data: injected dose (divided into three categories: < 100 MBq, 100–150 MBq, and ≥ 150 MBq), injected volume (divided into three categories: < 0.2 mL, 0.2–0.3 mL, and ≥ 0.3 mL), experience of preparer (divided into two categories: < 50 preparations, and ≥ 50 preparations) and experience of administrator (divided into two categories: < 50 administrations, and ≥ 50 administrations).

### Preoperative imaging protocol

Technetium-99 m radiolabeled albumin nanocolloid (from March 2011 to February 2019: Nanocoll, GE Healthcare, the Netherlands; from March 2019 to April 2021: Nanoscan, Radiopharmacy, Hungary) was administrated via an intra-tumoral injection, by a resident or an experienced nuclear medicine physician, either by palpation in palpable tumors or ultrasound-directed in nonpalpable tumors. An injected dose of approximately 120 MBq in a volume of 0.25 mL was administered in patients if the patient underwent surgery at the same day. Patients could also receive a dose of 240 MBq if surgical removal of the SLN was planned for the next day. Planar lymphoscintigraphy was performed at 15 min, 2 h (h), and sometimes 4 h post-injection (pi) intervals (see below). Focal accumulations in at least one axillar lymph node was defined as SLN. SLN nonvisualization was clinically classified as nonvisualization when no SLN was visualized on routine clinical lymphoscintigraphy.

### Reinjection

If planar lymphoscintigraphy showed SLN nonvisualization at 2 h pi, SPECT/CT imaging or a second periareolar injection of 120 MBq, followed by repeated planar lymphoscintigraphy 2 h later (i.e., 4 h after the initial injection), was performed.

### Statistical analysis

Patient, tumor and radiotracer characteristics were evaluated using descriptive statistics. Furthermore, each characteristics was tested for any association with SLN nonvisualization. Univariate logistic-regression models were used to examine the relationships between the different characteristics and SLN nonvisualization. Pearson Chi-Square exact test was used for categorical variables and the Mantel–Haenszel exact test was used for ordinal variables. Variables with a *P* value below 10% in the univariate analysis were included for the multivariable logistic-regression models. All statistical tests were two-tailed and a *P* value below 5% was considered statistically significant. Odds ratios (ORs) of significant risk factors are presented with calculation of 95% confidence interval (CI). All analysis were performed with IBM SPSS Statistics (version 26, IBM, USA).

## Results

### Preoperative lymphoscintigraphy

**Fig. 1:**
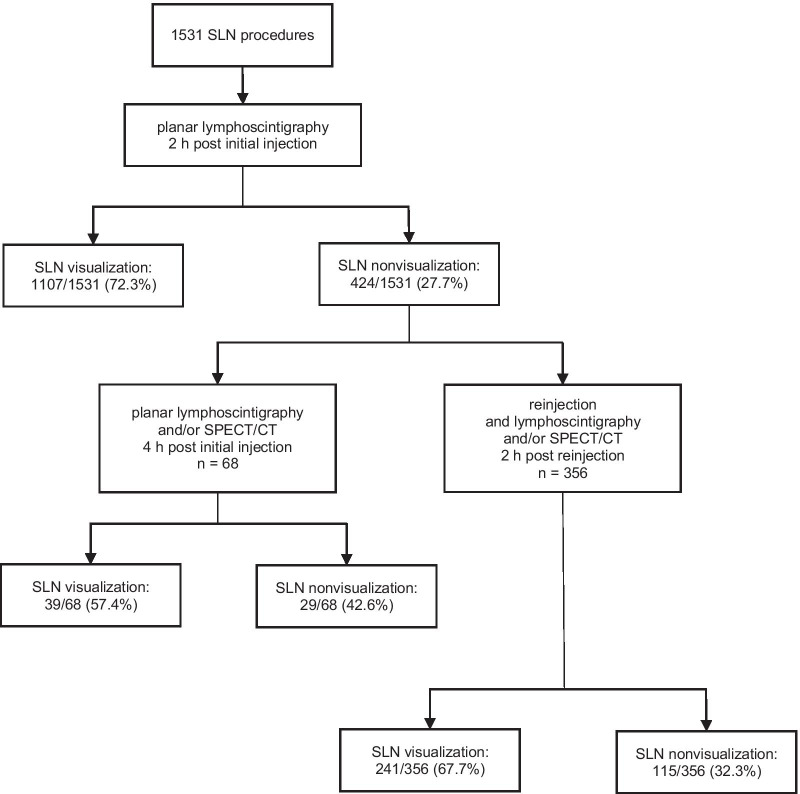
Flowchart of procedures with and without preoperative SLN visualization

A total of 1462 breast cancer patients, including 7 men and 37 female patients who underwent a second unilateral procedure due to a new malignancy, with a total of 1531 unique SLN procedures (i.e., 32 bilateral procedures, 1467 unilateral procedures), were enrolled in this study (see Fig. 1). Mean patient age was 59.6 years (SD 11.9 years) and the mean BMI was 27.9 kg/m^2^ (SD 5.8 kg/m^2^). Preoperatively, the SLN was visualized on planar lymphoscintigraphy at 2 h pi in 72.3% (1107/1531) of the SLN procedures and nonvisualization occurred in 27.7% (424/1531) of the SLN procedures (see Fig. 1). The visualization and nonvisualization of the SLN were 74.9% (1146/1531) and 25.1% (385/1531), respectively, at late lymphoscintigraphy 4 h pi.

### Reinjection and additional imaging after SLN nonvisualization

Out of the 424 SLN procedures with nonvisualization at 2 h pi, 356 SLN procedures did receive a periareolar reinjection of the radiotracer (see Fig. 1). After reinjection, 32.3% (115/356) of the SLN procedures had persistent SLN nonvisualization. In conclusion, the reinjection and additional imaging increased the visualization rate of the SLN to 90.6% (1387/1531).

### Risk factors of SLN nonvisualization after initial injection

Table 1 presents the number of SLN procedures with nonvisualization at late lymphoscintigraphy 4 h pi after the initial injection. The multivariable analysis showed that age ≥ 70 years (*P* < 0.001; OR: 2.27; 95% CI: 1.46–3.53), BMI ≥ 30 kg/m^2^ (*P* = 0.031; OR: 1.48; 95% CI: 1.04–2.12) and nonpalpable tumors (*P* = 0.004; OR: 1.54; 95% CI: 1.15–2.07) were independent predictors of SLN nonvisualization on lymphoscintigraphy at 4 h pi. Differences in tumor location, brand of radiopharmaceutical, injected dose, injected volume, experience of preparer and administrator did not lead to a significant increased risk for SLN nonvisualization.

**Table 1:** Results of multivariable analysis for risk factors of sentinel lymph node nonvisualization on lymphoscintigraphy after initial injection of the radiotracer

Characteristics	N	N of nonvisualization (%)	Univariate analysis	Multivariable analysis	
			*P*-value	Adjusted OR (95% CI)	*P*-value
Age (years)			< 0.001^b^		
< 50	364	57 (15.7)		1	
50–70	867	216 (24.9)		1.25 (0.85–1.82)	0.254
≥ 70	300	112 (37.3)		2.27 (1.46–3.53)	< 0.001
BMI (kg/m^2^)			0.003^b^		
< 25	363	83 (22.9)		1	
25–30	313	91 (29.1)		1.24 (0.87–1.77)	0.229
≥ 30	284	95 (33.5)		1.48 (1.04–2.12)	0.031
Unknown	571				
Tumor palpability			< 0.001^a^		
Palpable	938	199 (21.2)		1	
Nonpalpable	593	186 (31.4)		1.54 (1.15–2.07)	0.004
Tumor location			0.591^a^		
Medial/central	418	108 (25.8)			
Lateral	803	219 (27.3)			
Unknown	310				
Brand radiopharmaceutical			0.182^a^		
Nanocoll	1225	299 (24.4)			
Nanoscan	306	86 (28.1)			
Injected dose (MBq)			0.172^b^		
< 100	6	1 (16.7)			
100–150	1446	370 (25.6)			
≥ 150	79	14 (17.7)			
Injected volume (ml)			0.475^b^		
< 0.2	409	107 (26.2)			
0.2–0.3	1075	268 (24.9)			
≥ 0.3	47	10 (21.3)			
Experience of preparer (preparations)			0.390^a^		
< 50	621	149 (24.0)			
≥ 50	910	236 (25.9)			
Experience of administrator (procedures)			0.739^a^		
< 50	297	88 (29.6)			
≥ 50	470	134 (28.5)			
Unknown	764				

^a^Pearson Chi-Square exact test for categorical variables

^b^Mantel–Haenszel exact test for ordinal variables

### Risk factors of SLN nonvisualization after reinjection

None of the patient, tumor or tracer characteristics were significantly associated with SLN nonvisualization after a reinjection with the radiotracer (see Additional file 1: Table S1).

## Discussion

We found that SLN nonvisualization occurred in 27.7% of procedures on planar lymphoscintigraphy at 2 h after an intratumoral injection of technetium-99 m albumin nanocolloid. This number decreased to 25.1% on late lymphoscintigraphy at 4 h pi. Periareolar reinjection reduced the SLN nonvisualization rate to 9.4% of the total SLN procedures. In addition, we showed that the choice of radiotracer does not have an impact on the SLN nonvisualization rate.

To the best of our knowledge, this study is the first to examine the influence of experience of the preparer and administrator of the radiotracer on SLN nonvisualization, and we show that the experience of the preparer or administrator are not associated with SLN nonvisualization. This observation may contribute to the generally accepted view that there are no significant differences in interpretations made by radiology residents and those made by staff radiologist [8–10]. Our results support the notion that SLN detection on lymphoscintigraphy is a very robust technique, that does not depend on the experience of the preparer or administrator of the radiotracer.

We could not find any association between experience of the preparer of the radiotracer and SLN nonvisualization. This was also expected because the preparations of the radiotracers were in full accordance with the recommendation of the *“Guideline on current good radiopharmacy practice for the small-scale preparation of radiopharmaceuticals”* [11]. The results of this study do not show any association between injected dose or volume of the radiotracer and SLN nonvisualizations. Although the injected volume of tracer solution is a subject of controversy in literature, detection rates of SLN visualizations seem not to be affected by these solution volumes [12]. Tanis et al. showed that a higher amount of radioactivity is associated with less SLN nonvisualizations and recommended a dose of at least 100 MBq of the radiotracer [2]. In our study population, 99.6% of patients received a dose of 100 MBq or more. We could not find a significant association between the used doses and SLN non-visualization. However, the particle size (diameter ≤ 80 nm) of both used radiotracers radiotracers in this study is within the same range. Interestingly, initial studies with tilmanocept (particle size of 7 nm) showed very low SLN nonvisualizations, between 1.4 and 8.0% [13–15]. However, results from a small prospective, double-blinded, randomized clinical trial demonstrated no statistically significant difference, regarding SLN nonvisualization between nanocolloids and tilmanocept at 30 min lymphoscintigraphy [14]. Nevertheless, more research is needed to investigate which radiopharmaceutical (nanocolloids or tilmanocept) has a lower SLN nonvisualization rate.

We found that risk factors for SLN nonvisualization on lymphoscintigraphy at 4 h pi are age ≥ 70 years, BMI ≥ 30 kg/m^2^, and nonpalpable tumors. These risk factors are in accordance with findings of other studies. Increased age [2–7] and higher BMI [3–6] are well-known risk factors for SLN nonvisualization on lymphoscintigraphy. It has been hypothesized that replacement of lymph nodes by fatty tissue decreases the capacity of lymph nodes to retain the radioactive colloid [13] and that increased fatty tissue in elderly patients causes decreased lymphatic flow in the breasts [14].

Nonpalpable tumors are less known as a risk factor for SLN nonvisualization [6]. Deeper located tumor are more often labeled as nonpalpable tumors because they are less accessible by palpation. Anatomical studies have shown that the density of lymphatic vessels in the skin is greater compared to breast parenchyma [12]. This difference in density of lymphatic vessels is perhaps the reason why nonpalpable tumors are associated with an increased risk for SLN nonvisualization. Whether tumor location is a risk factor for SLN nonvisualization [3, 6] is still disputable. We and other studies could not find a significant effect of tumor location on SLN nonvisualization [2, 4, 5, 7].

In this study, we report an initial preoperative SLN visualization rate of 74.9%, which is in line with the literature [2–7]. The SLN visualization rate improved to 67.5% of the initial nonvisualized SLN after reinjection. This is comparable with Pouw et al. who found a SLN visualization rate of 62.1% after reinjection [15]. In our study population, the reinjection of the radiotracer increased the preoperative SLN visualization rate from 74.9% to 90.6%. Fortunately, however, the intraoperative SLN visualization rate is much higher, since some SLN are also detected by, e.g., combining nanocolloid detection with blue dye [16]. This study shows that reinjection is an adequate option to improve the SLN visualization rate for nuclear medicine departments which are flexible enough to apply an additional injection and imaging slots. Another interesting approach to reduce SLN nonvisualization may be a multisite injection technique [17]. Based on our findings, it may be of interest to evaluate in prospective studies the postulate that administration of both intratumoral and periareolar injections simultaneously to patients with non-palpable tumors and age ≥ 70 years or BMI ≥ 30 kg/m^2^ may reduce preoperative SLN nonvisualization.

The strength of this study is the large number of patients with lymphoscintigraphy data. In addition, our analysis included time corrected activity doses of the radiotracer, experience of the preparer and administrator, which was not studied before. However, this study has several limitations that need to be addressed. As patients received a fixed dose of the radiotracer (i.e., no correction for BMI), weight and height measurements were not available in all patients. Despite this limitation, the number of the patients in whom weight and height were registered had sufficient statistical power to examine the effect of BMI on SLN nonvisualization. Other limitations were that some characteristics of the tumor (stage, size) and lymph node (exact status, number of positive lymph nodes) were not available. These factors could be confounders, since some studies have indications that these factors are possible associated with SLN nonvisualization [2, 3, 6].

## Conclusions

This study shows that risk factors for SLN nonvisualization in breast cancer patients during preoperative lymphoscintigraphy are age ≥ 70 years, BMI ≥ 30 kg/m^2^ and nonpalpable tumors. Our results support the notion that SLN lymphoscintigraphy is a very robust technique, that does not depend on the experience of the preparer or administrator of the radiotracer used.

## Supplementary Information

**Additional file 1.** **Table S1.** Results of univariate analysis for risk factors for sentinel lymph node nonvisualization on lymphoscintigraphy after reinjection of the radiotracer.

## Data Availability

The datasets used and/or analyzed during the current study are available from the corresponding author on reasonable request.

## Abbreviations

SLN: Sentinel lymph node
BMI: Body mass index
