# Supplementary material for: Risk factors for nonvisualization of the sentinel lymph node on lymphoscintigraphy in breast cancer patients
Source: EJNMMI Res. 2021 Jun 9;11:54. doi: 10.1186/s13550-021-00793-8 (PMC8190239; doi:10.1186/s13550-021-00793-8)
Supplement: Supplementary file 1 — Additional file 1. Table S1. Results of univariate analysis for risk factors for sentinel lymph node nonvisualization on lymphoscintigraphy after reinjection of the radiotracer. [file 13550_2021_793_MOESM1_ESM.docx]

**Table S1. Results of univariate analysis for risk factors for sentinel lymph node nonvisualization on lymphoscintigraphy after reinjection of the radiotracer**

| Characteristics | N | N of nonvisualization (%) | P-value |
| --- | --- | --- | --- |
| Age (years)  <50  50–70  ≥70 | 56  198  102 | 20 (35.7)  60 (30.3)  35 (34.3) | 0.981^b^ |
| BMI (kg/m^2^)  <25  25–30  ≥30  Unknown | 78  86  88  104 | 19 (24.4)  31 (36.0)  27 (30.7) | 0.268^b^ |
| Tumor palpability  Palpable  Nonpalpable | 190  166 | 57 (30.0)  58 (34.9) | 0.320^a^ |
| Tumor location  Medial/central  Lateral  Unknown | 100  204  52 | 27 (27.0)  66 (32.4) | 0.341^a^ |
| Brand radiopharmaceutical  Nanocoll  Nanoscan | 274  82 | 88 (32.1)  27 (32.9) | 0.891^a^ |
| Injected dose (MBq)  <100  100–150  ≥150 | 8  340  8 | 6 (75.0)  106 (31.2)  3 (37.5) | 0.109^b^ |
| Injected volume (ml)  <0.2  0.2–0.3  ≥0.3 | 30  246  80 | 13 (43.3)  77 (31.3)  25 (31.3) | 0.382^b^ |
| Experience of preparer (preparations)  <50  ≥50 | 126  230 | 45 (35.7)  70 (30.4) | 0.308^a^ |
| Experience of administrator (procedures)  <50  ≥50  Unknown | 88  125  143 | 23 (26.1)  36 (28.8) | 0.669^a^ |

a Pearson Chi-Square exact test for categorical variables

b Mantel–Haenszel exact test for ordinal variables
